# Effect of deep neuromuscular block on the quality of early recovery after sleeve gastrectomy in obese patients: a randomized controlled trial

**DOI:** 10.1186/s12871-024-02465-1

**Published:** 2024-03-16

**Authors:** Wan-li Yang, Ya-ling Wen, Wen-mei Xu, Chi-liang Xu, Wen-qin Yin, Jing-yan Lin

**Affiliations:** 1 Department of Anesthesiology, Affiliated Hospital of North Sichuan Medical College, Nanchong, Sichuan 637000 China; 2 Department of Anesthesiology, North Sichuan Medical College, Nanchong, Sichuan 637000 China

**Keywords:** Obese, Deep neuromuscular block, Laparoscopic sleeve gastrectomy, Recovery

## Abstract

**Background:**

Deep neuromuscular block (NMB) has been shown to improve surgical conditions and alleviate post-operative pain in bariatric surgery compared with moderate NMB. We hypothesized that deep NMB could also improve the quality of early recovery after laparoscopic sleeve gastrectomy (LSG).

**Methods:**

Eighty patients were randomized to receive either deep (post-tetanic count 1–3) or moderate (train-of-four count 1–3) NMB. The QoR-15 questionnaire was used to evaluate the quality of early recovery at 1 day before surgery (T0), 24 and 48 h after surgery (T2, T3). Additionally, we recorded diaphragm excursion (DE), postoperative pain, surgical condition, cumulative dose of analgesics, time of first flatus and ambulation, post-operative nausea and vomiting, time of tracheal tube removal and hospitalization time.

**Main results:**

The quality of recovery was significantly better 24 h after surgery in patients who received a deep versus moderate block (114.4 ± 12.9 versus 102.1 ± 18.1). Diaphragm excursion was significantly greater in the deep NMB group when patients performed maximal inspiration at T2 and T3 (*P* < 0.05). Patients who underwent deep NMB reported lower visceral pain scores 40 min after surgery; additionally, these patients experienced lower pain during movement at T3 (*P* < 0.05). Optimal surgical conditions were rated in 87.5% and 64.6% of all measurements during deep and moderate NMB respectively (*P* < 0.001). The time to tracheal tube removal was significantly longer in the deep NMB group (*P* = 0.001). There were no differences in other outcomes.

**Conclusion:**

In obese patients receiving deep NMB during LSG, we observed improved QoR-15 scores, greater diaphragmatic excursions, improved surgical conditions, and visceral pain scores were lower. More evidence is needed to determine the effects of deep NMB on these outcomes.

**Trial registration:**

ChiCTR2200065919. Date of retrospectively registered: 18/11/2022.

## Introduction

During bariatric surgery, deep neuromuscular block (NMB) seems to provide surgeons with a better surgical field and wider operating space [1–4]. However, the clinical application of deep NMB is limited due to the prolonged recovery time of patients’ spontaneous breathing and the potential risk of residual paralysis; therefore, the use of deep NMB is still controversial [5, 6]. A systematic review showed that deep NMB improved surgical conditions and reduced post-operative pain compared with moderate NMB during bariatric surgery, whereas it fails to shorten the procedure duration [7]. However, it remains inconclusive whether deep NMB reduce post-operative complications and improves the quality of early recovery in bariatric surgery. Previous studies on the application of deep NMB in bariatric surgery have primarily focused on surgical conditions, post-operative pain, post-operative pulmonary function and the use of low CO_2_ pneumoperitoneum [1, 2, 4, 8]. However, these studies have yet to provide a comprehensive evaluation of the quality of early recovery in obese patients who underwent laparoscopic sleeve gastrectomy (LSG). As such, it remains unclear whether the reported benefits of deep NMB have translated into higher-quality early recovery.

Quality of post-operative recovery is a comprehensive concept that requires evaluation from both the perspective of healthcare professionals and the subjective experiences and emotions of patients. We chose the QoR-15 questionnaire as an appropriate assessment tool for evaluating the quality of early recovery [9]. The questionnaire was developed in 2013 by Stark et al. and has been confirmed by some studies to meet the requirements of appropriateness, reliability, validity, precision, acceptability, and feasibility [10–12]. A Chinese version of The QoR-15 questionnaire has also been developed and exhibits similar advantages to the English version [13]. The QoR-15 questionnaire assesses five dimensions: physical comfort, physical independence, psychological support, pain, and emotional state. We are confident that it can accurately evaluate the quality of early recovery in obese patients undergo LSG.

This trail was designed to evaluate whether deep NMB, as compared with moderate NMB, can enhance the quality of early recovery in obese patients undergoing LSG.

## Methods

### Ethics and registration

The Ethics Committee of the Affiliated Hospital of North Sichuan Medical College approved this single-center, randomized trial [2019ER338-1], which was registered in the Chinese Clinical Trials Registry [ChiCTR2200065919] before enrolment of the first patient. Patients were included in the study between November 2022 and October 2023. All methods were performed in accordance with relevant guidelines and regulations, and written informed consent was obtained from all patients before inclusion in the trial.

### Participants

Eligible patients were American Society of Anesthesiologists (ASA) physical status I-III patients with age 18 to 60 years, body mass index (BMI) > 28 kg m^− 2^, scheduled to undergo LSG, were recruited to participate in the study. Exclusion criteria were as follows: (1) neuromuscular disorders; (2) allergies to or contraindications for muscle relaxants, neuromuscular reversing agents, anesthetics, and narcotics; (3)pregnancy or lactation; (4) renal insufficiency; (5) chronic obstructive pulmonary disease GOLD classification 2 or higher; (6) clinical, radiographic, or laboratory findings suggestive of upper or lower airway infection; (7) congestive heart failure; (8) psychiatric illness inhibiting cooperation with the study protocol or possibly obscuring results; and (9) diaphragm with poor ultrasound visualization.

### Randomization and blinding

The eligibility for inclusion was assessed in the ward 1d before surgery and the first QoR-15 score was performed. All enrolled patients were randomized to either deep or moderate NMB groups based on a computer-generated random list. Surgeons, patients, ward nurses, and researchers were blinded to treatment allocation. The treatment allocation was concealed in sealed opaque envelopes. The attending anesthetic staff was not blinded because they had to maintain an adequate level of NMB according to the allocation of treatment.

### Anesthesia and study protocol

The surgical and anesthesia techniques were the same in both groups, except for the maintenance of NMB. According to the Good Clinical Research Practice recommendation, a post-tetanic count (PTC) of 1–3 was maintained in the deep NMB group (D group), and a train-of-four count (TOF) stimulation of 1–3 was maintained in the moderate NMB group (M group) [14]. NMB was monitored in the abducted left arm with a TOF Watch-SX acceleromyography device (Veryark-TOF, Guangxi, China) at the adductor pollicis muscle as recommended. Using forced air warming blankets, the central core temperature and skin temperature over the adductor pollicis muscle were maintained above 35.5℃ and 32℃, respectively [14]. All patients underwent surgery by one abdominal surgeon, who was experienced in LSG. Pneumoperitoneum was achieved and maintained at 14 mmHg by insufflation of CO_2_ through a Veress needle. Standard hemodynamic and respiratory monitoring established on arrival in the operating room consisted of electrocardiography, blood pressure, heart rate and bispectral index monitoring using a Philips BIS module system (BR-6000D, Hefei, China). BIS values were maintained between 40 and 60 throughout anesthesia. After 3 min of preoxygenation (100% oxygen by mask) in slight anti-Trendelenburg position, and dexamethasone (10 mg) intravenously, anesthesia was induced in all patients with 2.5 to 4.5 mg kg^− 1^ Propofol and an effect-site target-controlled infusion (TCI) of 3.0 to 4.5 ng ml^− 1^ remifentanil according to the pharmacokinetic model by Minto et al. [15]. Until the patient lost consciousness NMB was induced with 0.6 mg kg^− 1^ rocuronium. Anesthesia was maintained with 2–4 vol% sevoflurane in oxygen-air (50%/50%), 2.0–4.0 ng ml^− 1^ TCI of remifentanil and bolus doses of 10 mg rocuronium as required. Remifentanil, propofol, fentanyl and neostigmine doses were adjusted to lean body weight, rocuronium dosing was adjusted to ideal body weight [16]. When the second twitch of the TOF reappeared, NMB was reversed with a combination of neostigmine 0.05 mg kg-1 and glycopyrrolate 0.01 mg kg^− 1^ in both groups, and patients were extubated when the TOF ratio was > 0.9. Remifentanil infusion was stopped 15 min before the end of surgery. Simultaneously, fentanyl 4 µg kg^− 1^ was administered to provide initial post-operative analgesia.

Post-operative nausea and vomiting (PONV) were managed by intravenous infusion of ondansetron (8 mg) every 12 h, with an additional 4 mg provided upon the patient’s request. Post-operative pain was evaluated using a visual analogue scale (VAS), and a standard infusion of Lornoxicam (8 mg) was administered every 12 h. In cases where the VAS score was ≥ 4, intravenous Tramadol (100 mg) was administered.

### Outcome measures

The follow-up period began 40 min after surgery (T1) and lasted until 48 h after surgery. The primary outcome was the QoR-15 score, assessed at three time points:1 d before surgery (T0), 24 h post-surgery (T2), and 48 h post-surgery (T3), ranging from 0 to 150.

**Fig. 1:**
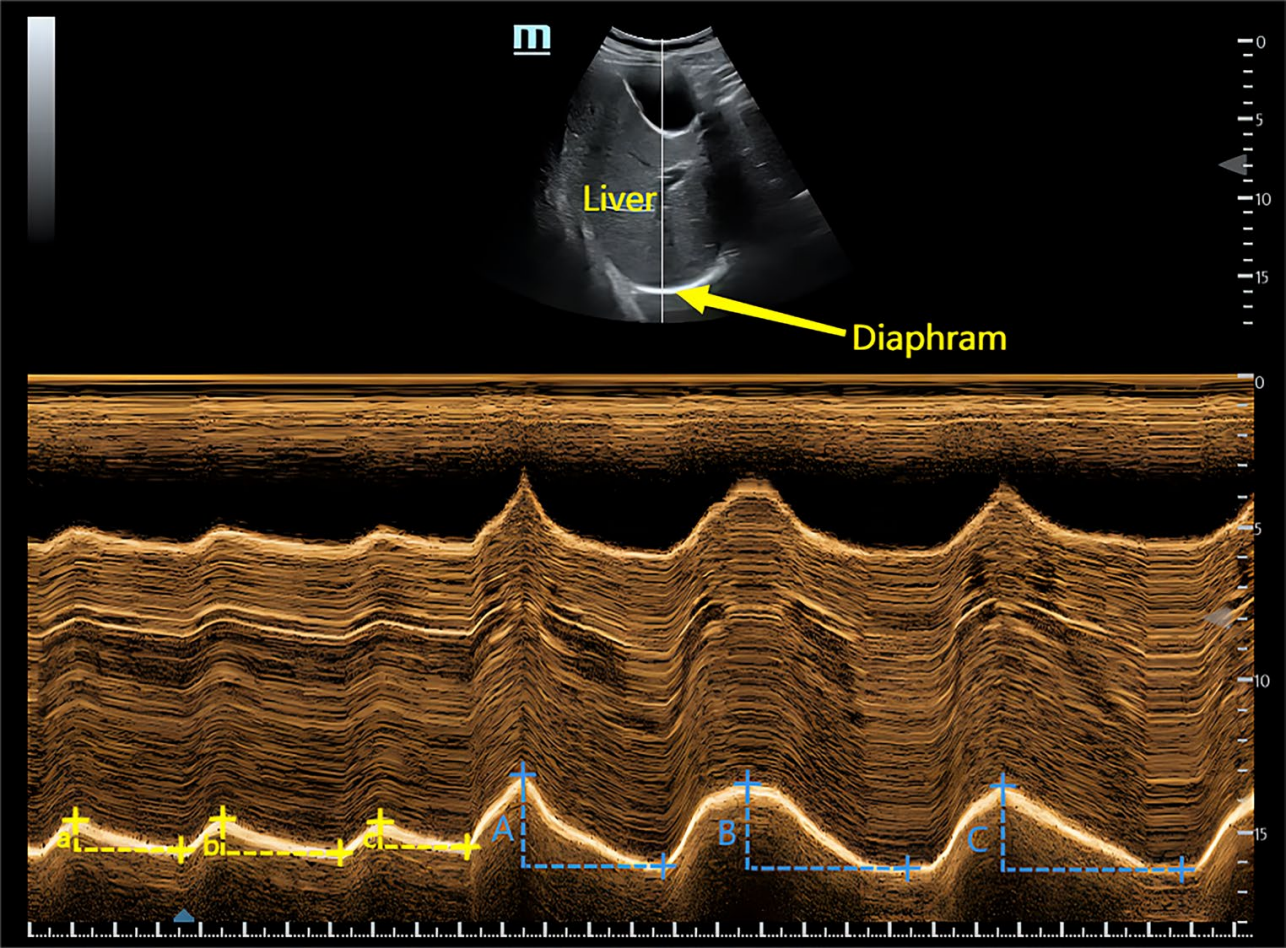
Measurement of diaphragm excursion. To minimize the measurement errors, the DE of each patient was measured three times in both normally and maximal respiration, and the average value was calculated for each state. Breath normally: (a + b + c)/3; Maximal inspiration: (A + B + C)/3

A physician who was experienced in diaphragmatic ultrasonography and blinded to treatment allocation performed the measurements of diaphragm excursion (DE) at T0, T1, T2, and T3. With the patient on the bed, a 3.5-5.0 MHz convex array probe was positioned in the subcostal region, between the right anterior axillary line and midclavicular line, using the liver as an acoustic window. The probe was adjusted for optimal orientation, and the ultrasound beam was aligned perpendicular to the posterior third of the diaphragm. In B-ultrasound mode, the liver, inferior vena cava, and diaphragm were visualized together, with the diaphragm appearing as a hyperechoic line between the lung and liver. Switching to M-mode, the diaphragm motion trajectory was observed along a selected measurement line, with diaphragm excursion (DM) measured as the vertical distance from the highest point to the baseline. To minimize the measurement errors, the DE of each patient was measured three times for both normally and maximal respiration, and the average value was calculated for each state.(Fig. 1) All patients were operated by one abdominal surgeon who scored the quality of the surgical condition using 5-point Leiden-Surgical Rating Scale in a 20-min interval during pneumoperitoneum [17]. Pain scores (visceral and incisional) at rest and during movement were recorded at T1, T2, and T3 using a VAS scale ranging from 0 to 10. Additional outcomes included cumulative analgesic dose, time of first flatus and ambulation, incidence of PONV, time of tracheal tube removal, and hospitalization time.

### Sample size and statistical analysis

The sample size was estimated by the QoR-15 scores 24 h after surgery, which was measured in 20 patients who underwent LSG. Considering a power of 80% with a type 1 error of 0.05, and a compliance rate of 90%, 94 patients were enrolled in this trial (47 patients per group). SPSS 26.0 (IBM Corp. Released 2021. IBM SPSS Statistics for Windows, Version 26.0; IBM Corp, Armonk, New York, USA) was used for statistical analysis. The Shapiro-Wilk test was performed to examine the assumption of normality. The Student’s *t* test was used to compare normally distributed continuous variables, and the Mann-Whitney *U* test was used to compare non-normally distributed continuous variables and ordinal variables. Categorical variables were compared using the χ2 test. QoR-15 and VAS scores were compared using the repeated measures ANOVA. DE was compared using the Wilcoxon rank sum test. All the measured values are presented as the number of patients (%), mean ± SD, mean (95% confidence interval), or median [IQR]. Statistical significance was set at *P* < 0.05.

## Results

**Fig. 2:**
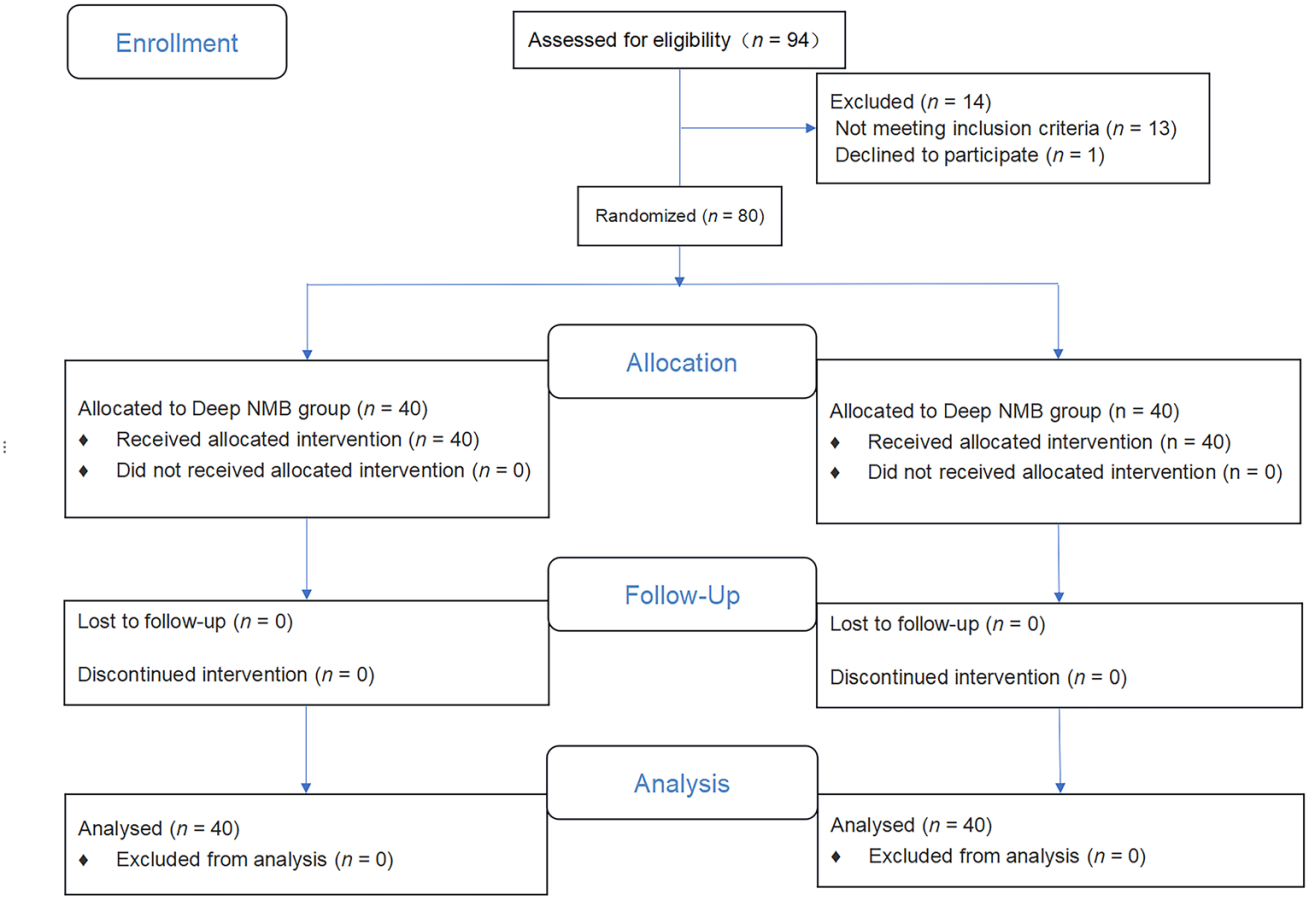
Consort flow diagram

Ninety-four patients were included in the study. Fourteen patients were excluded:13 because of poor ultrasound visualization of the diaphragm and one because of refusal to participate in the trial (Fig. 2). Data from 80 patients were analyzed (40 patients in each group) (Table 1).

**Table 1:** Patient characteristics

	M group (*n* = 40)	D group (*n* = 40)	P
Gender (male/female)	5/35	6/34	0.745
Age (years)	34 [31–38]	35 [30–40]	0.566
Height (cm)	163.25 ± 8.68	164.96 ± 8.94	0.388
Weight (kg)	93.10 ± 19.77	94.46 ± 20.05	0.728
LBW (kg)	51.59 ± 11.03	56.14 ± 11.48	0.810
IBW (kg)	54.93 ± 9.04	60.45 ± 9.94	0.534
BMI (kg m^− 2^)	34.34 ± 5.41	34.12 ± 4.96	0.849
Waistline (cm)	111.78 ± 14.96	111.33 ± 14.31	0.891
Hypertension (mmHg)	7/17.5%	7/17.5%	1.000
Type 2 diabetes	9/22.5%	7/17.5%	0.576
Dyslipidemia	27/67.5%	20/50%	0.112
Abdominal surgery history	25/62.5%	24/60%	0.818
OSAHS	7/17.5%	10/25%	0.412
ASA 1/2/3	0/22/18	0/24/16	0.644

Data are mean ± SD, medians [IQR] or number/%. LBW, lean body weight. IBW, ideal body weight. BMI, body mass index. OSAHS, Obstructive sleep apnea-hypopnea syndrome

### Primary outcome

**Fig. 3:**
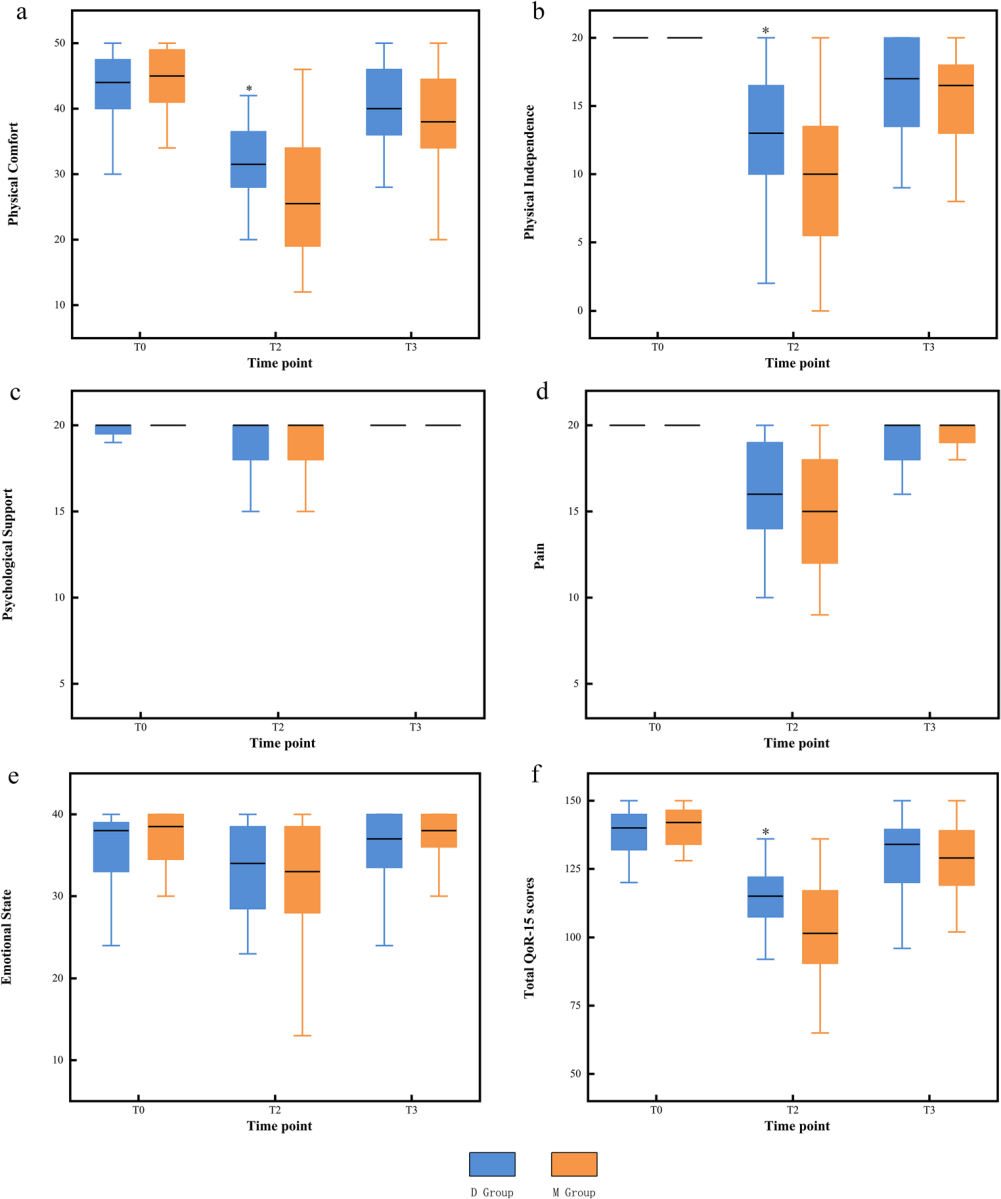
Each dimension and total of QoR-15 scores varies over time in the two groups. T0: 1d before surgery; T2: 24 h after surgery; T3: 48 h after surgery; QoR-15: Quality of Recovery-15 questionnaire. *Compared with M group the difference was significant at 0.05 level

The pre-operative QoR-15 scores were not significantly different between the two groups (*P* > 0.05). At T2, the D group had significantly higher QoR-15 scores than the M group (*P* = 0.001). In the D group, physical comfort and physical independence scores were significantly higher than in the M group (*P* < 0.05). No significant difference was observed between the two groups at T3 (*P* > 0.05; Fig. 3).

### Secondary outcomes

There was no statistically significant difference in DE between the two groups when the subjects breathed normally at all time points (*P* > 0.05). The DE of the D group was significantly greater than that of the M group when the patient performs maximal inspirations at T2 and T3 (*P* < 0.05). (Table 2)

**Table 2:** Diaphragm excursion at each time point

	M group	D group	P
Before surgery (cm)			
Breath normally	1.47 [1.30–1.85]	1.48 [1.17–1.77]	0.195
Maximal inspiration	4.18 [3.79–5.54]	4.68 [3.73–5.24]	0.686
40 min after surgery			
Breath normally	1.17 [1.06–1.26]	1.19 [1.05–1.51]	0.305
Maximal inspiration	2.33 [2.00-2.99]	2.63 [2.11–3.49]	0.037
24 h after surgery			
Breath normally	1.22 [1.05–1.50]	1.30 [1.15–1.41]	0.363
Maximal inspiration	3.01 [2.48–3.65]	3.53 [2.73–4.12]	0.030
48 h after surgery			
Breath normally	1.31 [1.16–1.54]	1.35 [1.17–1.54]	0.862
Maximal inspiration	3.58 [2.92–4.43]	3.89 [3.31–4.50]	0.275

Data are medians [IQR]. DE, diaphragm excursion; PACU, Post anesthesia care unit

Surgical rating scores were measured 112 times in the D group and 113 times in the M group. The frequency of measurements in each patient was similar in both groups (*P* > 0.05). Optimal surgical conditions were rated in 87.5% (98/112) and 64.6% (73/113) of all measurements during deep and moderate NMB, respectively (*P* < 0.001; Table 3).

**Table 3:** Measurements obtained during and after surgery

	M group (*n* = 40)	D group (*n* = 40)	P
Degree of NMB	TOF:1.5 [1.2-2.0]	PTC: 2 [1.6–2.4]	
Remifentanil (µg)	1284.9 ± 290.7	1270.6 ± 309.5	0.832
Fentanyl (µg)	225.0 [200.0-287.5]	237.5 [200.0-275.0]	0.845
Surgical rating score			
Frequency of measurements	3 [2–3]	3 [2–3]	0.877
Optimal (5)	73 (64.6%)	98 (87.5%)	0.012
Intra-abdominal pressure (mmHg)	13.6 [13.0–14.0]	13.0 [12.5–13.8]	0.067
Duration of surgery, min	118.8 ± 25.21	122.3 ± 30.28	0.458
Tracheal tube removal time (min)	44.5 [35.0-69.5]	50.0 [45.3–56.8]	0.001
Duration in the PACU (min)	62.5 [55.0–85.0]	62.5 [60.0–70.0]	0.521
The first ambulation time (h)	8.8 [4.3–17.3]	6.8 [4.8–12.5]	0.821
The first flatus time (h)	33.8 [22.7–61.0]	27.3 [18.9–64.1]	0.427
PONV	24 (60%)	17 (42.5%)	0.179
Tramadol (mg)	0.0 [0.0-100.0]	0.0 [0.0-100.0]	0.546
Hospitalization time (h)	116.5 [96.3-139.3]	98.0 [96.0-120.0]	0.281

Data are mean ± SD, number of patients (%) or median [IQR]. PONV, post-operative nausea and Vomiting

Incisional pain at rest did not show a statistically significant difference between the two groups at any time points (*P* > 0.05). Visceral pain at rest at T1 differed significantly between the two groups (*P* = 0.002). At T2, pain scores were not significantly different between the two groups (*P* > 0.05). At T3, both visceral and incisional pain during movement in the M group were significantly higher than those in the D group (*P* < 0.05). (Table 4)

**Table 4:** Pain score at each time point

	M group	D group	P
40 min after surgery			
Incisional pain at rest	0.8 (0.3–1.2)	0.7 (0.3-1.0)	0.732
Visceral pain at rest	3.9 (3.3–4.4)	2.9 (2.4–3.3)	0.006
24 h after surgery			
Incisional pain at rest	1.4 (0.7-2.0)	0.8 (0.5–1.2)	0.215
Incisional pain during movement	2.5 (1.7–3.3)	2.1 (1.6–2.5)	0.351
Visceral pain at rest	2.1 (1.4–2.9)	1.7 (1.2–2.1)	0.297
Visceral pain during movement	3.8 (3.1–4.6)	3.0 (2.4–3.6)	0.059
48 h after surgery			
Incisional pain at rest	0.5 (0.1–0.9)	0.3 (0.1–0.5)	0.342
Incisional pain during movement	1.6 (1.1–2.1)	0.9 (0.5–1.2)	0.020
Visceral pain at rest	0.7 (0.2–1.1)	0.6 (0.3–0.9)	0.923
Visceral pain during movement	2.2 (1.6–2.8)	1.2 (0.8–1.5)	0.005

Data are mean (95% confidence interval)

There were no differences between the two groups in analgesic consumption, time of first flatus and ambulation, PONV, hospitalization time, intra-abdominal pressure, duration of surgery, and PACU stay. The TOF ratio was > 0.9 at extubation in all patients. Compared to the M group, the time of tracheal tube removal was significantly longer in group D (*P* = 0.001). (Table 3)

## Discussion

This study used the QoR-15 questionnaire for the first time to evaluate whether the deep NMB improves the quality of early recovery in obese patients undergoing LSG. The most important finding is that, deep NMB was associated with a better quality of early recovery than moderate NMB in obese patients who underwent LSG. Second, deep NMB can prompt post-operation recovery of diaphragmatic function. Third, compared to moderate NMB, deep NMB improved the surgical condition and alleviated post-operative pain.

A recent study updated the minimum clinically important difference for QoR-15 as 6 points to conclude that an effect exists [18]. Our study demonstrated a clinically significant difference of 12.0 at T2 between the two groups. This result suggests that in obese patients who underwent LSG, deep NMB can improve the quality of early recovery compared to moderate NMB. In our study, deep NMB promoted the recovery of diaphragmatic function, improved the surgical condition and alleviated post-operative pain. Based on these advantages, deep NMB improve the quality of early recovery by improving the two dimensions of physical comfort and physical independence in QoR-15.

DE as a clinical assessment tool for diaphragmatic dysfunction in obese patients has not been extensively studied compared to other patient populations, such as ICU patients. While specific studies on DE in obese patients are limited, measuring DE can still offer insights into diaphragmatic function in this population. In a study by Leticia BALTIERI et al. [19]. diaphragmatic excursion was assessed using radiographic images in patients undergoing open bariatric surgery to evaluate the functional status of the diaphragm. Similarly, in the study by Marcela Cangussu Barbalho-Moulim et al. [20]. radiographic measurements of diaphragmatic excursion were employed to evaluate the functional status of the diaphragm in subjects after open bariatric surgery. In a case report by Martine Ferrandiere MD et al. [21]. ultrasound was utilized to measure DE after spinal anesthesia in an obese patient, revealing a 30% reduction in diaphragmatic excursion. However, the implementation of noninvasive positive pressure ventilation led to an improvement in diaphragmatic excursion and overall respiratory function. Given these findings and recognizing the potential of ultrasound in evaluating diaphragmatic function in obease patients, we employ ultrasound to measure DE as a means of assessing the diaphragmatic function status of our subjects.

In this study, we observed varying degrees of post-operative impairment in diaphragm function in both groups compared with pre-operative levels. The DE during maximal inspiration at T1 and T2 was significantly lower in the M group than in the D group, indicating that deep NMB might be beneficial for diaphragmatic function. Upper abdominal laparoscopic procedures may result in transient diaphragmatic dysfunction, primarily due to temporary neuronal deactivation induced by traction on afferent nerve fibers distributed within the diaphragm or peritoneum during pneumoperitoneum establishment [22]. On the other hand the increase in visceral injury will lead to an increase in noxious stimulation of the visceral nerve afferent fibers, which will inhibit the reflex activity of the phrenic nerve and reduce the central phrenic nerve impulse, resulting in the deterioration of diaphragm function [23]. Thus, we speculate that deep NMB could reduce noxious stimulation during surgery, which can aid in the recovery of diaphragm function. However, it is worth to note that when using ultrasound to measure the diaphragmatic excursion in obese patients, due to the interference of increased tissue depth and adipose tissue, as well as the limitation of pain on the activity of diaphragm, this may affect the accuracy and reliability of ultrasonic measurement of diaphragmatic excursion. Consequently, the clinical applicability of ultrasonic measurement of DE in this population requires further confirmation through future studies.

Post-operative pain, particularly during movement, can hinder early ambulation in patients and negatively impact the recovery of gastrointestinal function, ultimately compromising physical comfort and independence. In the present study, we observed that post-operative pain during movement was significantly higher than that at rest in both groups. Additionally, visceral pain was significantly greater than incisional pain, which may be attributed to the larger gastric body incision following LSG. We also found that the D group had significantly less visceral pain at T1 and significantly less pain during movement at T3 than the M group. This may be attributed to an improved surgical field, resulting in reduced tissue and organ trauma inflicted by the surgeon during the procedure [24, 25]. A recent meta-analysis involving four randomized controlled trials reported that deep NMB helps improve surgical conditions and reduces post-operative pain in patients undergoing laparoscopic bariatric surgery; however, it fails to shorten the procedure duration, which is in agreement with our study results [7]. The improved surgical conditions can enable the surgeon to operate more precisely and avoid accidental tissue and organ trauma. Multiple studies have shown similar results [1, 3, 17, 26, 27]. LSG entails removing the greater curvature of the stomach within a confined abdominal space, followed by meticulous suturing of the wound edge. These procedures inevitably injure the tissues and organs around the gastric body, necessitating a broad and stable surgical area. An optimal surgical field facilitates precise surgical procedures and potentially reduces the risk of iatrogenic injury, thereby alleviating post-operation pain and diaphragm dysfunction to improve the quality of early recovery.

In our study, neostigmine was selected as the reversal agent for both groups, as opposed to sugammadex. Consequently, there was a prolonged extubation time observed in group D compared to group M. While sugammadex efficiently reverses deep NMB induced by rocuronium, we did not choose it as a reversal agent due to its high cost and absence of coverage by medical insurance in China [28]. This decision aimed to prevent an additional economic burden on patients. Importantly, our choice of the reversal agent and its dosage remained in line with current recommendations [29]. The judicious administration of neostigmine, guided by objective neuromuscular monitoring, proves beneficial in eliminating residual paralysis and reducing post-operative respiratory complications [30]. 

This study had several limitations. First, in our study, ultrasound visualization of the diaphragm was hindered by the excessive abdominal fat in patients. Additionally, obesity-induced increases in intra-abdominal pressure and alterations in respiratory mechanics further impact diaphragmatic function. Consequently, relying solely on ultrasound for assessing diaphragm function in obese patients may lack accuracy and reliability. Therefore, exploring alternative methods for efficiently and effectively evaluating diaphragm function in this patient population warrants further research and exploration. Second, the conclusions of the study relate only to obese patients undergoing LSG. Whether this is the case with other laparoscopic procedures should be evaluated in future studies. Third, Due to the high cost of sugammadex, neostigmine was selected as the reversal agent in both groups. Consequently, group D exhibited a slower recovery from NMB compared to group M. Fourth, we did not collect data on post-operative complications during our study, thus preventing determination of whether varying depth of NMB influence their incidence.

In conclusion, in patients receiving deep NMB during LSG, we observed improved QoR-15 scores, greater diaphragmatic excursions, improved surgical conditions, and visceral pain scores were lower. However, it is worth noting that deep neuromuscular blockade significantly prolongs the extubation time, which should be closely monitored in clinical application. More evidence is needed to determine the effect of deep neuromuscular blockade on these outcomes.

## Data Availability

No datasets were generated or analysed during the current study.

## Abbreviations

QoR-15: Quality of Recovery-15
NMB: neuromuscular block
PTC: post-tetanic count
TOF: train-of-four count
DE: diaphragm excursion
PACU: Post anesthesia care unit
ASA: American Society of Anesthesiologists
BMI: Body mass index
GOLD: Global Initiative for Chronic Obstructive Lung Disease
BIS: Bispectral index
TCI: Target-controlled infusion
VAS: Visual analogue scale
PONV: Postoperative nausea and vomiting
SD: Standard deviation
IQR: Interquartile range
CO2: carbon dioxide
